# Is hemoglobin concentration affected by sepsis in the acute phase?

**DOI:** 10.1186/cc11948

**Published:** 2013-03-19

**Authors:** G Jansma, H Buter, RT Gerritsen, EC Boerma

**Affiliations:** 1 Medical Centre Leeuwarden, the Netherlands

## Introduction

In the acute phase of sepsis several potential mechanisms may change the hemoglobin (Hb) concentration. On the one hand, endothelial activation may lead to increased vascular permeability and fluid sequestration to the interstitium, leading to hemoconcentration. On the other hand, degradation of the glycocalyx has been reported [1]. Shedding of this carbohydrate-rich layer with an estimated thickness of 0.2 to 0.5 µm may lead to a substantial increase of the intravascular space, and thus to decrease of Hb concentration [2]. The aim of this study is to determine whether there is a decrease in Hb in the acute phase of sepsis.

## Methods

In this single-center retrospective analysis we identified patients with sepsis as the primary reason for non-elective ICU admission from a standard patient database. Patients who fulfilled the international criteria of sepsis and organ failure during ICU admission were included in the sepsis group (S-group). The control group was formed by patients with other non-elective reasons for ICU admission (C-group). Exclusion criteria were (recent) bleeding, surgery in the last 6 weeks, chronic renal failure (creat >177 μmol/l, or hemodialysis), untreated chronic anemia, pregnancy, polytrauma, age <18, hematologic or metastasized malignancies, cardiac arrest, and use of bone marrow suppressive drugs. Laboratory data were collected from blood samples, prior to in-hospital i.v. fluid therapy. In order to detect a difference in Hb concentration of 0.23mmol/l, we anticipated a sample size of 283 per group, based on a standard deviation (SD) of 1.2, α = 0.05 and β = 0.8. Data are expressed as mean ± SD.

## Results

We included 296 patients in the S-group and 320 in the C-group. The difference in Hb between the S-group and C-group was not significant (8.76 ± 1.18 mmol/l vs. 8.93 ± 1.16 mmol/l, *P *= 0.07). After correction for a number of confounders, using a multivariate regression analysis, we observed a significant difference in Hb of -0.23 mmol/l in the S-group in comparison with the C-group (*P *= 0.01).

## Conclusion

At first presentation, prior to in-hospital i.v. fluid therapy, Hb concentration in patients with sepsis is significantly lower in comparison with controls; however, the difference is very small, without the existence of anemia.
